# Role of albumin and prealbumin in assessing nutritional status and predicting increased risk of infectious complications during childhood cancer treatment

**DOI:** 10.3389/abp.2024.13693

**Published:** 2024-11-15

**Authors:** Anna Milaniuk, Katarzyna Drabko, Agnieszka Chojęta

**Affiliations:** ^1^ Department of Hematology, Oncology and Transplantology, Medical University of Lublin, Lublin, Poland; ^2^ Department of Pediatric Laboratory Diagnostic, Medical University of Lublin, Lublin, Poland

**Keywords:** albumin, prealbumin, children, cancer, malnutrition

## Abstract

**Introduction:**

Proper nutrition in patients with cancer is important for preventing treatment complications and achieving remission. Malnutrition in these patients leads to reduced production of essential structural proteins.

**Purpose:**

The aim of the study was to assess the role of albumin and prealbumin in assessing the nutritional status of cancer patients and in predicting an increased risk of infectious complications during treatment.

**Patients and Methods:**

The study included 40 pediatric patients with newly diagnosed cancer and 30 healthy children serving as controls. Prealbumin, albumin, and C-reactive protein (CRP) levels and the upper arm muscle area (UAMA) were measured before and after treatment in children with cancer and compared with the control group to evaluate nutritional status. Additionally, we assessed associations between these parameters and the incidence of infectious complications during cancer treatment in patients with anthropometric malnutrition, as well as associations with an increased risk of malnutrition related to inflammation before treatment.

**Results:**

At baseline, patients with cancer had lower prealbumin and albumin levels (p< 0.001), higher CRP levels (p < 0.001), and lower UAMA percentiles (p = 0.0245) compared with controls. Cancer treatment resulted in an increase in prealbumin and albumin levels (p < 0.001) and a reduction in CRP levels (p < 0.001), with no change in UAMA (p = 1.000). Prealbumin deficiency was more common than albumin deficiency before and after cancer treatment. Median prealbumin and albumin levels tended to increase with an increasing UAMA percentile range, but these differences were not significant (p> 0.05). The incidence of infectious complications during treatment in patients with risk factors for inflammation-related malnutrition was similar to that in patients with pre-existing anthropometric malnutrition without inflammation (p = 1.000). In a univariable logistic regression model including prealbumin and albumin deficiency, as well as low UAMA percentile, albumin deficiency before treatment was shown to be a significant predictor of 3 or more infectious episodes during treatment (p = 0.02).

**Conclusion:**

Albumin and prealbumin deficiency may predict the risk of malnutrition associated with inflammation in patients with cancer. Hypoalbuminemia may predict an unfavorable course of treatment complicated by frequent infections in these patients.

## Introduction

Thanks to advances in pediatric cancer treatment, the number of childhood cancer survivors is growing. In recent years, a reduction in childhood cancer mortality has been observed in Europe, with Poland being one of the countries with the lowest leukemia mortality rates (Bertuccio et al., 2020). On the other hand, the long duration of cancer treatment, the use of cytostatic and biologic drugs, as well as radiotherapy and surgery, may lead to treatment-related complications. The most common complications are infections, which can be life-threatening in immunosuppressed patients, and thus reduce cancer survival (Esbenshade et al., 2023; Zawitkowska et al., 2019). As a result, cancer remains the third leading cause of death (after accidents and injuries) in the pediatric population (Cunningham et al., 2018).

Supportive care in cancer patients is important to prevent serious complications or to mitigate their effects (Esbenshade et al., 2023). One component of supportive care is to improve the nutritional status of patients. Malnutrition at diagnosis increases the risk of mortality during and after treatment (Barr and Stevens, 2020). Proper nutritional status in cancer patients promotes remission and improves patient comfort (Rogers and Barr, 2020). Therefore, it is important to identify patients at risk for malnutrition.

The assessment of nutritional status in children with cancer should include common anthropometric measures such as body weight and body mass index, as well as baseline body composition evaluated by mid-upper arm circumference and triceps skinfold thickness, or by more advanced tests such as bioelectrical impedance analysis and dual-energy X-ray absorptiometry (Ladas et al., 2016). Anthropometric nutritional status can also be determined by the upper arm muscle area (UAMA) index, which represents lean body mass and is calculated using the Frisancho formula (Frisancho, 1990).

Increased catabolism and energy expenditure in cancer patients results in reduced production of structural proteins (Bauer et al., 2011; Co-Reyes et al., 2012; Laviano et al., 2005; Sala et al., 2004; Soeters et al., 2021; Romano et al., 2022). These metabolic processes manifest clinically as muscle wasting and poor muscle function both in adult and pediatric patients (Joffe et al., 2019; Shachar et al., 2016; Cederholm et al., 2017). Malnutrition in cancer is a type of disease-related malnutrition with inflammation, where the degree of disease-induced metabolic response determines the rate of catabolism and the onset of clinically significant malnutrition (Cederholm et al., 2017; Viani et al., 2020). Patients with precachexia are at risk for malnutrition due to the inflammatory response induced by the underlying disease (Sala et al., 2004).

Given that low serum protein levels may predict inflammatory malnutrition, we aimed to assess the protein status of children with newly diagnosed cancer and the effect of pretreatment albumin and prealbumin deficiency on the incidence of infectious complications during treatment (Cehreli et al., 2019; Luo et al., 2019).

## Patients and methods

### Study design

This observational, prospective, single-center study was conducted in pediatric patients with newly diagnosed cancer who were hospitalized between October 2019 and January 2022 in the Pediatric Department of Hematology, Oncology and Transplantology at University Hospital in Lublin, Poland. In total, 40 children with cancer were included. The results of the study group were compared with the results of the control group, which was recruited from volunteers. The control group included children who were not receiving specialized care for chronic diseases, were not taking any chronic medications, and had no evidence of active infection on enrollment.

Median age at the time of cancer diagnosis was 11.29 years (IQR, 5.25–13.27), and the median age of the control group was 6.5 years (IQR, 4.65–9.96). Most patients were male (67.5%), had a diagnosis of hematological malignancy (75%), and received low-intensity or intermediate-intensity treatment (55%). The median follow-up was 39.93 weeks (IQR, 26.57–48.64), and the median time between the end of cancer treatment and study measurements was 6.07 weeks (IQR, 3.21–11.89). The baseline characteristics of the study are summarized in Table 1.

**TABLE 1:** Characteristics of 40 children with cancer.

Characteristics of 40 children with cancer	
Sex Female Male	n (%) 13 (32.5) 27 (67.5)
Age, years	Median (Q1-Q3) 11.29 (5.25–13.27) Range 2.08–17.67
Primary diagnosis Hematological malignancies Acute lymphoblastic leukemia Acute myeloid leukemia Non-Hodgkin’s lymphoma Hodgkin’s lymphoma Solid tumors Central nervous system tumors Wilms tumor Soft tissue sarcoma Ewing sarcoma Germ cell tumors	n (%) 30 (75.0) 15 2 5 8 10 (25.0) 2 2 3 2 1
Intensity of treatment^a^ Low/intermediate High	n (%) 22 (55.0) 18 (45.0)

^a^ Treatment intensity was defined according to risk group classification or disease stage as follows: SR, standard risk; IR, intermediate risk, stage I, II (low/intermediate intensity of treatment), HR, high risk, stage III, and IV (high intensity of treatment).

In all patients, the levels of visceral serum proteins (prealbumin and albumin) and C-reactive protein (CRP) were assessed, and anthropometric measurements were performed before and after cancer treatment to evaluate nutritional status as well as associations between these parameters and the incidence of infectious complications during cancer treatment. As malnutrition is a risk factor for infectious complications, we assessed the incidence of complications in patients with anthropometric malnutrition and those with an increased risk of malnutrition related to inflammation before treatment. Patients with anthropometric malnutrition met the following criteria: low UAMA percentile range and low albumin and/or low prealbumin and normal CRP levels, whereas patients with normal/high UAMA and low albumin and/or low prealbumin and elevated CRP levels were suspected of malnutrition with inflammation.

The clinical and demographic data of patients were obtained from hospital medical records. In all patients, cancer was diagnosed based on the International Classification of Childhood Cancer ver. 3 (Steliarova-Foucher et al., 2005). Patients received standard treatment according to the type and stage of cancer.

Detailed clinical, demographic, and social characteristics of study participants were reported previously (Milaniuk and Drabko, 2024).

Informed consent was obtained from parents or legal guardians of each participant included in the study.

#### Ethics approval

This study was conducted in accordance with the principles of the Declaration of Helsinki. Approval was granted by the Ethics Committee of the Medical University of Lublin (26 Sep 2019/No KE-0254/278/2019).

### Biochemical assessment of serum protein levels

Blood samples were collected from patients after an overnight fast during routine sampling for laboratory measurements. Serum prealbumin levels were determined by enzyme-linked immunosorbent assay (IDK^®^ prealbumin ELISA, Immunodiagnostik AG, Germany) in samples previously stored at −20°C. Albumin and CRP levels were determined immediately after sample collection. Albumin deficiency was defined as a level of 3.5 g/dL or lower and prealbumin deficiency as a level of 0.2 g/L or lower. Prealbumin levels of less than 0.1 g/L indicated severe deficiency. CRP levels greater than 0.5 mg/L indicated the risk of inflammation.

### Assessment of anthropometric protein-energy undernutrition

In this study, UAMA was used to anthropometrically assess protein-energy undernutrition (PEU) in cancer patients. It was calculated using the Frisancho formula: UAMA (cm^2^) = ${\left[\text{MUAC}-\left(\text{TSFT}*\pi \right)\right]}^{2}∕\left(4*\pi \right)$ , where MUAC is the mid-upper arm circumference and TSFT is the triceps skinfold thickness.

UAMA percentiles were classified into the following categories: low muscle (<5th percentile), below average (5th to <15th percentile), average (15th to <85th percentile), above average (85th to <95th percentile), and high muscle (≥95 percentile) according to age- and sex-adjusted growth charts (Frisancho, 1990).

### Assessment of infectious complications

The medical records of participants were reviewed for the history of the following infectious complications during treatment: respiratory infections, gastrointestinal inflammation, stomatitis, soft tissue inflammation, fever of unknown origin (FUO), and sepsis. Standardized International Classification of Diseases, Tenth Revision (ICD-10) diagnostic codes were used in the analysis.

### Statistical analysis

Patient characteristics were evaluated using descriptive statistics, categorical variables were described as numbers and percentages, and numerical variables were described as mean, standard deviation, median, quartiles, and range. Differences between groups were assessed using Fisher, χ^2^, Mann-Whitney, Wilcoxon, Kruskal-Wallis. A single-variable logistic regression analysis was used to assess prealbumin and albumin deficiency and low percentile ranges of the UAMA as predictors of infectious complications during treatment. For each explanatory variable OR was calculated. A p-value of <0.05 was considered significant. All statistical analyses were performed using R version 4.1.1.

## Results

### Protein levels and UAMA percentile ranges in cancer patients and controls

Albumin and prealbumin levels were significantly lower in cancer patients than in controls. In the cancer group, albumin and prealbumin levels were higher after vs. before treatment (Table 2). In contrast, CRP levels were significantly higher in cancer patients than in controls. Cancer treatment led to a significant reduction in CRP levels (Table 2).

**TABLE 2:** Protein concentrations in patients before and after cancer treatment and in controls.

	Proteins					
	Prealbumin g/L	p-value	Albumin g/dL	p-value	CRP mg/L	p-value
Study group n = 40 before treatment	0.08 (0.07–0.15) 0.02–0.34	<0.001^a^	4.11 (3.87–4.44) 3.06–5.07	<0.001^a^	0.68 (0.07–3.31) 0.01–23.13	<0.001^a^
Study group n = 40 after treatment	0.18 (0.15–0.25) 0.1–0.38		4.58 (4.42–4.73) 3.92–5.11		0.09 (0.03–0.26) 0.01–2.09	
Controls group n = 30	0.17 (0.15–0.22) 0.12–0.47	<0.001^b^	0.17 (0.15–0.22) 0.12–0.47	<0.001^b^	0.03 (0.03–0.03) 0.03–0.27	0.001^b^

Data are presented as median (Q1-Q3) and range, and the differences in parameters were calculated using the Wilcoxon test.

^a^ Patients before vs. after cancer treatment.

^b^ Patients before cancer treatment vs. control group.

Abbreviations: CRP, C-reactive protein.

Regarding UAMA, there was a significantly higher number of patients with low percentiles in the cancer group compared with the control group. There were no differences in the distribution of UAMA percentiles before vs. after treatment in cancer patients (Table 3).

**TABLE 3:** Distribution of UAMA percentiles in patients before and after cancer treatment and in controls.

	UAMA percentile			p-value^a^	p-value^b^
	<5–15th	15–85th	>85th		
Study group n=40 before treatment	11 (27.5)	22 (55.0)	7 (17.5)	0.0245^c^	1.0000^d^
Study group n=40 after treatment	11 (27.5)	22 (55.0)	7 (17.5)		
Controls group n=30	1 (3.3)	22 (73.3)	7 (23.3)		

Data are presented as number and percentage (%). The differences in parameters were calculated using the Fisher test^c^ and χ^2^ test^d^.

^a^ Patients before cancer treatment vs. control group.

^b^ Patients before vs. after cancer treatment.

UAMA, upper arm muscle area.

### Protein levels and UAMA values in patients with hematological malignancies and solid tumors

There were no significant differences in protein levels and UAMA values between group of patients with hematological malignancies and those with solid tumors (neither before nor after cancer treatment (p > 0.05). However, the comparison within groups demonstrated that in patients with hematological malignancies prealbumin and albumin concentrations were significantly higher after treatment that before. Similat observation was made in patients with solid tumours, but it regard only to albumin concentrations (Table 4).

**TABLE 4:** Protein concentrations and UAMA values in patients before and after cancer treatment according to cancer type.

Type of cancer	Prealbumin g/L	p-value	Albumin g/dL	p-value	UAMA (cm^2^)	p-value
Hematological malignancies, n = 30 before treatment	0.08 (0.06–0.1) 0.02–0.2	<0.001	4.11 (3.86–4.49) 3.06–5.07	0.0012	24.14 (16.59–36.02) 10.26–65.4	0.0803
Hematological malignancies, n = 30 after treatment	0.18 (0.16–0.25) 0.1–0.38		4.57 (4.38–4.8) 3.92–5.11		25.12 (19.2–35.42) 12.15–62.82	
Solid tumors n = 10 before treatment	0.14 (0.09–0.19) 0.03–0.34	0.4922	4.19 (3.91–4.42) 3.68–4.64	0.0098	21 (19.51–26.3) 16.1–53.48	0.8127
Solid tumors n = 10 after treatment	0.15 (0.13–0.18) 0.1–0.26		4.6 (4.48–4.64) 4.38–4.74		21.63 (20.09–28.48) 15.56–55.81	

Data are presented as median (Q1-Q3) and range. Differences in parameters were calculated the Wilcoxon test.

Abbreviations: UAMA, upper arm muscle area.

### Assessment of prealbumin and albumin deficiency

In patients with cancer, prealbumin deficiency was more common before vs. after treatment (p < 0.001). Before treatment, it was noted in 35 patients (87.5%), of whom 23 (57.5%) had severe prealbumin deficiency. After treatment, prealbumin deficiency was reported in 25 patients (67.5%). None of the cancer patients had severe prealbumin deficiency after treatment. Albumin deficiency was found in 5 patients (12.5%) before treatment, while it was not observed in any of the patients after treatment (p = 0.0547). Before treatment, 21 patients (52.5%) had elevated CRP levels, as compared with 8 patients (20%) after treatment (p = 0.0053).

The median prealbumin level across all UAMA percentile ranges was less than 0.2 g/L. However, patients with UAMA below the 85th percentile had the median prealbumin level below 0.1 g/L, what indicated a severe deficiency. The median albumin level for all UAMA percentile ranges was greater than 3.5 g/dL. In addition, median albumin and prealbumin levels tended to increase with an increasing UAMA percentile, but the difference was not significant (p > 0.05). There was no association between CRP levels and UAMA percentile range (Table 5).

**TABLE 5:** Pretreatment prealbumin, albumin, and CRP levels depending on the UAMA percentile range.

UAMA percentile range	Prealbumin g/L	p-value	Albumin g/dL	p-value	CRP (mg/L)	p-value
<5–15th n = 11	0.08 (0.06–0.14) 0.02–0.2	0.1013	4.08 (3.74–4.35) 3.32–4.61	0.4759	0.13 (0.03–0.3) 0.03–1.34	0.2002
15–85th n = 22	0.08 (0.06–0.1) 0.03–0.21		4.05 (3.88–4.48) 3.06–5.07		0.03 (0.03–0.15) 0.01–2.09	
>85th n = 7	0.14 (0.11–0.18) 0.07–0.34)		4.42 (4.07–4.58) 3.36–4.67		0.1 (0.1–0.7) 0.03–1.43	

Data are presented as median (Q1-Q3) and range. Differences in parameters were calculated using the Kurskal-Wallis test.

Abbreviations: CRP, C-reactive protein; UAMA, upper arm muscle area.

### Associations between nutritional status at diagnosis and infectious complications

**FIGURE 1:**
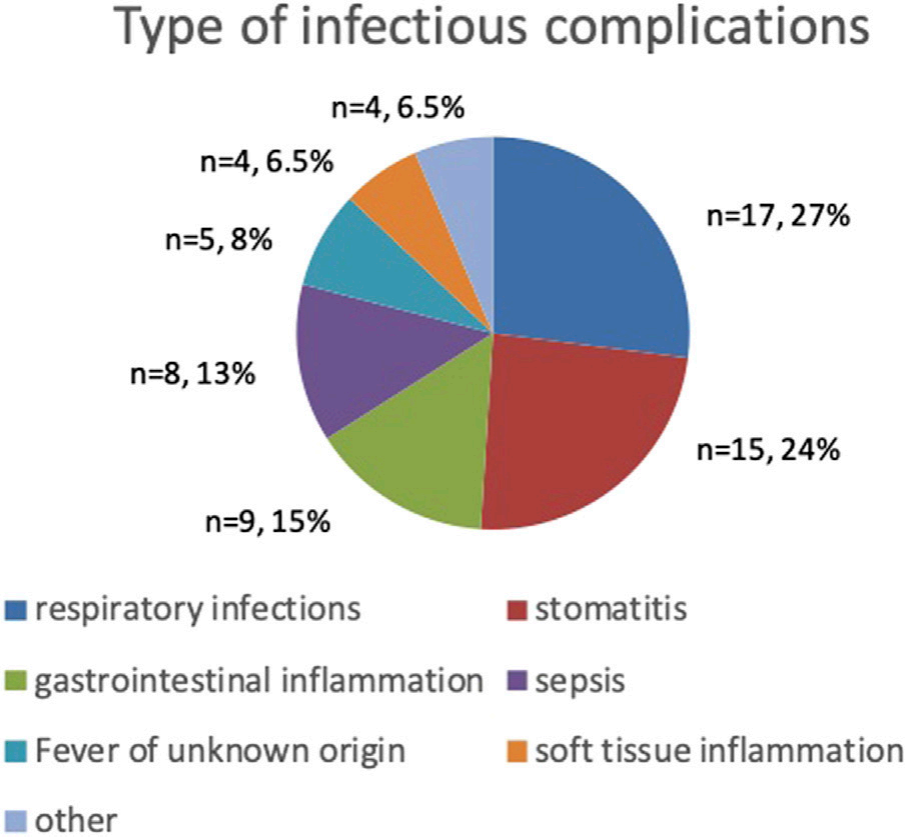
Type and frequency of infectious complications.

The most common infectious complications during the course of treatment affected respiratory system (n = 17) with almost half of them being pulmonary mycosis (n = 8), and oral cavity (n = 15) with stomatitis reaching grade III and IV of severity (n = 5). The third most common infectious complication were gastrointestinal tract infections (n = 9). Sepsis was diagnosed in 8 patients and FUO occurred in 5. The remaining infections regard to soft tissue infections, asymptomatic COVID-19 and varicella (Figure 1). Severe mucosal inflammation (typhlitis, stomatitis) required total parenteral nutrition in 2 patients. In the remaining cases, oral nutritional interventions were used.

Infectious complications occurred in 23 patients with cancer (57.5%), with 3 or more infectious episodes reported in 11 patients (27.5%). The incidence of complications was influenced by tumor type, with 3 or more episodes occurring significantly more often in patients with hematological malignancies than in those with solid tumors (63.2% vs. 40%; p = 0.0381). Factors such as sex, intensity of treatment, and patient age had no effect on the incidence of infectious complications (p > 0.05).

Pretreatment prealbumin and albumin levels were slightly lower in patients with infectious complications during cancer treatment compared with patients without complications (Table 6). However, these differences were not significant, except borderline significance for prealbumin. Patients with hypoalbuminemia were significantly more likely to have 3 or more infectious episodes (Table 7).

**TABLE 6:** Pretreatment prealbumin and albumin levels according to the occurrence of infectious complications.

Infectious complications	Prealbumin g/L	p-value	Albumin g/dL	p-value
Yes n = 23	0.08 (0.05–0.1) 0.02–0.2	0.0504	0.12 (0.08–0.19) 0.03–0.34	0.073
No n = 17	0.12 (0.08–0.19) 0.03–0.34		4.38 (4.08–4.52) 3.06–5.07	

Data are presented as median (Q1–Q3) and range. Differences in parameters were calculated using the U Mann-Whitney test.

**TABLE 7:** Distribution of prealbumin and albumin deficiency according to the number of infectious episodes.

	Infectious complications		
	0–2 episodes n = 29	3–5 episodes n = 11	p-value
Prealbumin deficiency			
Yes n = 35	24 (68.6)	11 (31.4)	0.2975
No n = 5	5 (100.0)	0 (0.0)	
Albumin deficiency			
Yes n = 5	1 (20.0)	4 (80.0)	0.0152
No n = 35	28 (80.0)	7 (20.0)	

Data are presented as number (percentage) of patients. Differences in parameters were calculated using the Fisher test.

### Infectious complications in patients with anthropometric malnutrition and risk of malnutrition with inflammation

The incidence of infectious complications during cancer treatment was compared between patients with anthropometric malnutrition without inflammation and those at risk of malnutrition due to inflammation. Only 4 patients (10%) met the diagnostic criteria for malnutrition without inflammation, while 15 patients (37.5%) were at risk for malnutrition due to inflammation. The remaining 21 patients with cancer (52.6%) did not meet any of the criteria to be included in either subgroup. We found no differences in the incidence of infectious complications between subgroups, and over 50% of patients in both subgroups had 3 or more infectious episodes (Table 8).

**TABLE 8:** Incidence of infectious complications in patients with anthropometric malnutrition and patients at risk for malnutrition with inflammation.

Infectious complications	Patients with anthropometric malnutrition n = 4	Patients at risk for malnutrition with inflammation n = 15	p-value
Yes	3 (75.0)	9 (60.0)	1.000
No	1 (25.0)	6 (40.0)	
Infectious complications			
	0–2 episodes	3–5 episodes	1.000
Patients with anthropometric malnutrition n = 4	1 (25.0)	3 (75.0)	
Patients at risk for malnutrition with inflammation n = 15	6 (40.0)	9 (60.0)	

Data are presented as number (percentage). Differences in parameters were calculated using the Fisher test.

### Effect of protein deficiency and low UAMA on the incidence of complications during cancer treatment

In a univariable logistic regression model including prealbumin and albumin deficiency, as well as low UAMA percentile, pretreatment albumin deficiency was shown to be a significant predictor of 3 or more infectious episodes during cancer treatment (p = 0.02) (Table 9).

**TABLE 9:** Univariable logistic regression analysis of prealbumin and albumin deficiency, as well as low UAMA percentile, as predictors of infectious complications during treatment.

Variable	Estimate	OR	LCI	UCI	p-value
Prealbumin deficiency	16.786	19,499,705.588	0.000	NA	0.992
Albumin deficiency	2.773	16.000	1.994	341.07	0.02
Low UAMA percentile	1.161	3.194	0.713	14.71	0.126

Odds ratio (OR), and lower (LCI) and upper (UCI) 95% confidence intervals for main risk factors.

Abbreviations: NA, not available; UAMA, upper arm muscle area.

## Discussion

This study highlights the importance of identifying malnutrition in children with cancer. We present an analysis of the protein and anthropometric status of protein malnutrition in cancer, which indicates the risk of infectious complications in malnourished patients and those at risk of malnutrition.

Previous studies confirmed that cancer patients have lower blood protein levels than healthy individuals and in addition that protein deficiency affects the treatment course and survival of these patients (Cehreli et al., 2019; Luo et al., 2019; McLean et al., 2020; Sanches et al., 2015; Kuvibidila et al., 2000). In our study, prealbumin and albumin levels were also significantly lower in children at the time of cancer diagnosis compared with healthy control. Moreover, blood protein levels increased after cancer treatment, which may indicate a metabolic switch to anabolic processes in cancer remission.

Protein in the diet is very important for cancer patients, whose metabolic rate is particularly high. Therefore, supportive cancer care should include nutritional management of malnourished patients. For this reason, the Polish Society for Pediatric Clinical Nutrition and the Polish Society for Pediatric Oncology and Hematology have developed guidelines for assessing the clinical risk of malnutrition in children with cancer and the indications for implementing nutritional interventions before and during treatment in patients at risk of malnutrition and malnourished patients (Budka-Chrzęszczyk et al., 2024). The enrichment of meals with foods that are high in protein and calories is a basic principle in the choice of diet for children with cancer. However, during cancer treatment, children’s taste and smell preferences often change, and treatment with cytostatic drugs is associated with vomiting and nausea, making it difficult to follow dietary recommendations. Patients at risk of permanent malnutrition during treatment should therefore be treated with nutritionally complete and energy-rich enteral formulas, this form of management provides uninterrupted nutritional support. In some cases, patients who have developed gastrointestinal mucosal complications may have difficulty with oral intake, and special situations such as acute pancreatitis and bowel obstruction may require temporary parenteral nutrition. The indications for nutritional interventions developed by the above Polish societies are supported by literature data (Bauer, et al., 2011; Ladas et al., 2016).

In our study group, up to 87% of children were found to be protein deficient before treatment. The results of our study did not confirm significant differences in protein status and the incidence of PEU when comparing the group of patients with solid tumours and hematological malignancies both before and after treatment. In contrast, the results of the analysis showed that there was an increase in protein levels after treatment in both tumour groups, with significantly higher levels of prealbumin and albumin in the hematological malignancies and significantly higher levels of albumin in the solid tumour group.

In our study, prealbumin deficiency was more common than albumin deficiency before treatment and was present in most patients. Prealbumin (transthyretin) is a transport protein for thyroid hormones. Serum prealbumin levels below 0.1 g/L are considered to indicate a significant risk of malnutrition (Keller, 2019; Beck and Rosenthal et al., 2002; Bharadwaj et al., 2016). In our study, 67.5% of patients had prealbumin deficiency after treatment, but the deficiency was not severe. We also observed that achieving higher prealbumin concentrations after treatment occurred in patients with hematological malignancies, who are at lower risk of malnutrition due to their tumour type. Additionally, most patients with hematological malignancies received low and intermediate intensity treatment due to their low risk group classification (standard risk or-intermediate risk), while most patients with solid tumours received high intensity treatment due to their high stage of disease. Compared with albumin, prealbumin has a short half-life of only 2 days. Due to its favorable amino acid composition, prealbumin can be used to assess changes in the patient’s diet, such as insufficient caloric intake or dietary improvement (Keller, 2019; Beck and Rosenthal et al., 2002; Bharadwaj et al., 2016). In our study, a lower percentage of patients with prealbumin deficiency after treatment may indicate an improvement in the nutrition of cancer patients in remission and thus predict an improvement in the nutritional status of patients. However, this hypothesis cannot be confirmed due to the limited duration of the study and the lack of monitoring of nutritional status and prealbumin levels in cancer survivors.

Literature data report a different prevalence of hypoalbuminemia at diagnosis in children with cancer, ranging from 7.4% to 45.8% (McLean et al., 2020; Leraas et al., 2023; Tandon et al., 2015; Yaprak et al., 2021). In our study, albumin deficiency was found in only 12.5% of children before treatment and in no children after treatment. Therefore, albumin was shown to be less useful than prealbumin in predicting malnutrition due to inflammation.

Our study highlights the measurement of UAMA as a simple but important tool for assessing lean body mass, particularly muscle mass, which is a key component of metabolic and immune health. The prevalence of anthropometric malnutrition according to UAMA in our patients did not change during the observation period. A low UAMA percentile was reported in 27.5% of cancer patients at diagnosis, and this rate remained stable until the end of treatment. Iniesta et al. (Revuelta Iniesta et al., 2019) reported a lower prevalence of PEU in their study group including 64 children with cancer. The highest prevalence of 14% was found at baseline and decreased to 10% at 3 months and to 12.5% at 24 months of follow-up. None of the patients had PEU at 30 and 36 months after diagnosis (Revuelta Iniesta et al., 2019). In our study, we did not confirm a significant relationship between PEU and the levels of selected proteins. However, we found that patients with normal and high UAMA percentiles had higher serum prealbumin and albumin levels than patients with low UAMA. In contrast, Yaprak et al. (2021) reported an association between anthropometric malnutrition in children with cancer and prealbumin deficiency. The rates of low serum prealbumin levels on admission and at follow-up were 54.3% and 39.4%, respectively. Low prealbumin levels were reported in 73% of patients with malnutrition vs. 42% of those without (p = 0.007). In contrast, Gürlek Gökçebay et al. (2015) highlighted significant changes in prealbumin levels following dietary intervention in patients diagnosed with anthropometric malnutrition. At 3 months of follow-up, there was a significant decrease in prealbumin levels in patients with vs. those without malnutrition (p = 0.04), while no significant reduction in the proportion of patients with malnutrition was reported. At 6 months, the proportion of patients with malnutrition was significantly lower than at baseline (p = 0.006) (Gürlek Gökçebay et al., 2015). These results indicate that it is possible to modify the UAMA parameter in those patients who have improved their diet, but that its improvement and increase from deficient values to normal values can be achieved over a longer period of time after completion of oncological treatment. However, this hypothesis also cannot be confirmed due to the limited duration of the study and the lack of monitoring of nutritional status and prealbumin levels in cancer survivors.

Malnutrition can make a person more susceptible to infection, which in turn increases the body’s need for energy and also contributes to the onset or worsening of malnutrition (Katona and Katona-Apte, 2008). In the inflammatory condition, protein synthesis processes in the liver correlate inversely with increases in CRP, so visceral protein deficiencies are often found in disease states where acute phase proteins are elevated (Evans et al., 2020). Proteins play an important role in supporting the immune system, and their deficiency can adversely affect immune function. They are essential for the proper functioning of immune cells, including T and B lymphocytes, and influence the production of cytokines that regulate immune responses. An adequate supply of protein is also necessary for the proper functioning of the complement system. In addition, proteins play a role in wound healing and a deficiency can interfere with tissue repair (Morales et al., 2023).

The incidence of infectious complications in cancer is influenced by disease- and treatment-related factors, such as bone marrow suppression, as well as by patient-related factors, such as inadequate nutritional status (Pedretti et al., 2023). Consequently, every patient with cancer is at risk for developing infectious complications during cancer treatment, which leads to a delayed completion of cancer treatment, increased risk of infection-related mortality, and cancer treatment failure. Among the infectious complications analysed in our patient group, some were serious and life-threatening (sepsis, FUO, fungal infections, stomatitis in stages III and IV, typhlitis). However, due to small sample size we did not analyze patients grouped according to infections severity, but we assessed the impact of prealbumin and albumin deficiency and PEU on the incidence of infectious complications during cancer treatment. We found a significantly higher number of infectious complications in patients diagnosed with hematological malignancies compared with those with solid tumors. Our findings are in line with the study by Zajac-Spychala et al. (2021). In our study, the incidence of infectious complications during treatment in patients with risk factors for inflammatory malnutrition was similar to that in patients with pre-existing anthropometric malnutrition without inflammation. We also found that patients at risk for malnutrition with inflammation were more than 3 times more likely to have known anthropometric malnutrition without inflammation. Although the diagnosis of malnutrition based on protein assessment is not recommended (Evans et al., 2021), our findings confirm that they can be used to identify inflammation leading to malnutrition that increases the risk of infectious complications during cancer treatment to the same extent as the presence of malnutrition itself.

We found lower pretreatment prealbumin and albumin levels in patients with infectious complications, with borderline statistical significance shown for prealbumin. In addition, pretreatment prealbumin deficiency was present in all patients who had more than 3 infectious episodes during treatment. This finding may support the role of proteins, particularly prealbumin, in assessing changes in nutritional status in children with cancer and in recognizing the need to implement supportive nutritional measures during cancer treatment. Liang et al. (2018) reported a significant increase in total protein, albumin, and prealbumin levels after nutritional intervention in children with acute lymphoblastic leukemia during induction treatment. They also showed a lower incidence of infection during induction treatment in patients who received nutritional therapy (Liang et al., 2018). Although most our patients had prealbumin deficiency and hypoalbuminemia was less common, albumin deficiency was shown to be a significant predictor of 3 or more episodes of infectious complications during treatment in our patients. The role of hypoalbuminemia as a negative prognostic factor for treatment outcome was demonstrated by other authors (McLean et al., 2020; Leraas et al., 2023; Tandon et al., 2015; Sonowal and Gupta, 2012).

The study has its strengths and limitations. The strengths include prospective design which enables to collect data in unified way and interdisciplinary approach of the study that combines clinical, nutritional and, in some cases, immunological perspectives relevant to supportive care during oncological treatment of childhood cancer. One of its limitations is the small sample size, which may have affected the statistical significance of the results. However, the number of patients included reflects the capacity of the department where the study was conducted at the time of the study. We are also aware that the study group was heterogeneous regarding diagnosis but subjecting to analyzis only patients with one type of cancer would make the study group even more smaller. The treatment approach is also different, as patients with hematological malignancies received steroid therapy. However, this is a standard treatment for this type of cancer, so we could not avoid its possible influence on the study results. We did not analyse the effect of nutritional interventions on the biochemical results obtained in this study, but we believe that the questions raised will encourage further research.

## Conclusions

The presence of protein-energy malnutrition in children with cancer at the time of diagnosis increases the risk of infectious complications during treatment. In our study, albumin and prealbumin deficiencies were shown to indicate the risk of malnutrition related to inflammation in patients with childhood cancer. Hypoalbuminemia may be useful for identifying cancer patients who are at risk for an adverse treatment course, complicated by frequent infections. Implementing nutritional interventions will be beneficial in patients with PEU and in those with suspected malnutrition with inflammation. Proper nutrition in cancer improves nutritional status and reuduces the risk of infectious complications during treatment.

## Data Availability

The original contributions presented in the study are included in the article/supplementary material, further inquiries can be directed to the corresponding author.
